# Fatty Liver Education Promotes Physical Activity in Vulnerable Groups, Including Those With Unhealthy Alcohol Use

**DOI:** 10.1016/j.gastha.2023.09.012

**Published:** 2023-10-05

**Authors:** Shyam Patel, Rebecca G. Kim, Amy M. Shui, Catherine Magee, Maggie Lu, Jennifer Chen, Michele Tana, Chiung-Yu Huang, Mandana Khalili

**Affiliations:** 1 Division of Gastroenterology and Hepatology, Department of Medicine, University of California, San Francisco, San Francisco, California; 2 Division of Gastroenterology and Hepatology, Zuckerberg San Francisco General Hospital and Trauma Center, San Francisco, California; 3 Department of Epidemiology and Biostatistics, University of California, San Francisco, San Francisco, California

**Keywords:** Exercise, Lifestyle Modification, Alcohol-Associated Liver Disease, Nonalcoholic Fatty Liver Disease, Underserved Populations, Steatotic liver disease

## Abstract

**Background and Aims:**

Fatty liver disease (FLD), alcohol-associated and metabolically associated, often coexists. Increase in physical activity is associated with metabolic health and decreased FLD. We aimed to identify factors associated with physical activity and its improvement following FLD education in a racially diverse, vulnerable population.

**Methods:**

From February 19, 2020 to December 30, 2022, 314 adults with FLD at safety-net hepatology clinics in San Francisco were surveyed at baseline, immediately after FLD education, and at 6-month follow-up. After collecting clinical and sociodemographic data, logistic regression (adjusted for age, sex, and race/ethnicity) assessed factors associated with physical activity at baseline and its improvement following education.

**Results:**

Participant characteristics in those without vs with any physical activity were median age 49 vs 55 years, 64% vs 56% female, 66% vs 53% Hispanic race/ethnicity, 75% vs 55% obese, and 30% vs 22% consumed heavy alcohol, respectively. On multivariable analysis, older age was the only significant factor associated with physical activity at baseline (relative risk ratio 1.37 per decade increase, 95% confidence interval [CI] 1.07–1.75). Hispanic (vs non-Hispanic) participants had a significantly higher odds of improvement in physical activity (vs no change) 6 months after education (odds ratio 2.36, 95% CI 1.27–4.39). Among those with suboptimal or no physical activity at baseline, participants who consumed heavy alcohol (vs no drinking) had a significantly higher likelihood of achieving optimal physical activity following education (relative risk ratio 1.98, 95% Cl 1.05–3.74).

**Conclusion:**

Despite social and structural barriers, FLD education increased uptake of physical activity in vulnerable populations, especially among Hispanic individuals and those consuming heavy alcohol. Implementation of patient-centered education is important for FLD management.

## Graphical abstract

**Figure:**
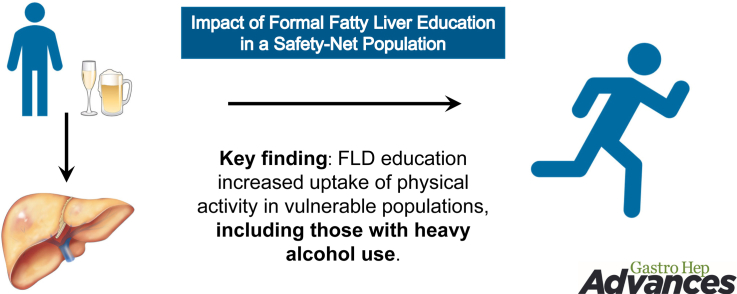

## Introduction

Fatty liver disease (FLD) describes the excess deposition of fat in the liver and is an umbrella term for metabolically associated fatty liver disease (MAFLD) and alcohol-associated liver disease (ALD). Patients deemed vulnerable, including socio-economically disadvantaged and minority groups, are disproportionately affected by both MAFLD and ALD and are at an increased risk for experiencing health disparities.^1^ Moreover, MAFLD and ALD often coexist. Epidemiological studies have suggested that MAFLD and ALD may share overlapping mechanistic pathways. For example, while a majority of patients who report heavy drinking develop fat in the liver, only 35% progress to advanced ALD, further suggesting that factors beyond alcohol could be leading to disease progression in this population.^2^ Indeed, among patients with ALD, metabolic risk factors like obesity are independently associated with both steatosis and cirrhosis.^3^ Moreover, obese patients with a history of heavy alcohol use have a higher propensity for developing steatosis and cirrhosis compared to their normal-weight counterparts.^4^

Most recently, models have shown that disease progression likely converges, sometimes even synergistically, through the metabolic pathways of insulin resistance, lipid dysregulation, oxidative stress, inflammation, and fibrogenesis.^5^^,^^6^ As we are beginning to understand the parallel nature of MAFLD and ALD pathophysiology, there is also a growing consensus that these conditions should be treated collectively. Currently, the primary treatment for MAFLD, in addition to managing metabolic abnormalities, is lifestyle modification. This includes increasing physical activity, improving dietary habits, weight loss, and alcohol cessation. Accordingly, evidence has shown that lifestyle modification can reduce hepatic adiposity^7^^,^^8^ and restore liver function,^9^ highlighting the importance of promoting healthy lifestyle changes, like the adoption of physical activity, in these patients.^10^^,^^11^

An increase in physical activity, irrespective of weight loss, has been associated with metabolic health and reduced FLD.^12^ Health education programs can facilitate the adoption of lifestyle modification, and these interventions have been linked to improved clinical outcomes in several associated chronic conditions, including cardiac disease^13^ and diabetes mellitus.^14^ Importantly, there are known social and structural barriers to adopting physical activity that are prevalent in vulnerable populations.^1^ Despite this, there is no study thus far that has evaluated the impact of a patient-centered educational program on changes in physical activity among a diverse, vulnerable population with FLD.

Therefore, the objectives of this study were to identify factors associated with physical activity at baseline and the impact of implementing formal FLD education on improvement in physical activity in a racially diverse and vulnerable population.

## Methods

### Study Population

This prospective study included 314 adult patients (aged ≥18 years) receiving care for FLD (76% with MAFLD, 21% with MAFLD and ALD, and 3% with ALD) at hepatology clinics in the San Francisco safety-net healthcare system from February 19, 2020 to December 30, 2022.^15^ Diagnosis of FLD and the ability and willingness to consent were required to participate in the study. Participants with a severe medical or psychiatric condition that prevented the completion of study activities, along with the inability or unwillingness to consent, were excluded from the study. Following informed consent, enrolled patients were surveyed before and after receipt of formal education on FLD and its management. This study was approved by the Institutional Review Board of the University of California, San Francisco and Zuckerberg San Francisco General Hospital.

### Study Intervention

Patients meeting eligibility criteria participated in a formal 60-minute FLD education session either in-person or remotely via video conference conducted by a designated hepatology Nurse Practitioner using PowerPoint slides. There was a maximum of 10 participants per session. The content of the curriculum consisted of FLD epidemiology, diagnosis, progression, prevention, management options, and lifestyle modifications, including information on diet, alcohol abstinence, and physical activity recommendations.^16^^,^^17^ The education session was conducted with the help of certified medical interpreters for non-English speakers as needed.

### Data Collection and Survey Design

Patients were surveyed before and immediately after education and at 6 months following education. Sociodemographic information at baseline was collected.^18^^,^^19^ The National Institute on Alcohol Abuse and Alcoholism questionnaire was used to categorize alcohol use.^20^ We assessed physical activity duration and intensity using a questionnaire that was developed based on clinical recommendations^16^^,^^17^ and assessed physical activity duration as none, ≥150 minutes/wk, and <150 minutes/wk and intensity as light (eg, walking leisurely, stretching, vacuuming, or light yard work), moderate (eg, fast walking, aerobics class, strength training, swimming gently), and vigorous activity (eg, stair machine, jogging or running, tennis, racquetball, pickleball, or badminton). Patient’s FLD knowledge, beliefs toward FLD, and barriers to lifestyle modifications were assessed similarly to previous studies using the Health Behavior Framework.^21^, ^22^, ^23^ The Health Behavior Framework domains evaluated were as follows: (1) FLD knowledge, (2) beliefs about FLD (with subdomains of perceived severity, treatment efficacy, self-efficacy to discuss FLD, perceived susceptibility to disease risk, and stigma), (3) lifestyle barriers to FLD management, (4) motivation to adhere to lifestyle modifications, and (5) medical mistrust. Clinical history and laboratory data were captured through manual electronic health record (EHR) review.

Participants were compensated $25 for participating in the informational session and answering all survey material. For non-English speakers, all surveys were translated into Spanish, the most prevalent language spoken outside of English in our population, and certified medical interpreters were used for other languages as needed.

### Sociodemographics, Clinical, and Laboratory Data Definitions and Measures

Demographic information consisted of age, sex, race/ethnicity, and social determinants of health, including marital status, birth country, primary language, education level, annual estimated income, employment status, housing stability, and number of individuals in a household. Using the National Institute on Alcohol Abuse and Alcoholism questionnaire,^20^ alcohol use was grouped into 3 categories: none, moderate (≤1 drink/d for women and ≤2 drinks/d for men), and heavy (>moderate).

Liver disease severity was evaluated by presence of advanced fibrosis and levels of aspartate transferase and alanine transaminase. Advanced fibrosis including cirrhosis was defined by contour nodularity of the liver [without (N = 13, 13%) or with splenomegaly or venous collaterals (N = 31, 31%)] on imaging, magnetic resonance elastography liver stiffness measurement > 4.5 kilopascals (N = 16, 16%), or a histologic fibrosis stage of F3-4 (N = 41, 40%). Clinical characteristics including etiology of liver disease were identified under medical history and problem list in the EHR. Body mass index information was also extracted from the EHR and race-adjusted as follows: normal <25 kg/m^2^ (<23 kg/m^2^ if Asian/Pacific Islander [API]), overweight 25–29.9 kg/m^2^ (23–27.4 kg/m^2^ if API), and obese ≥30 kg/m^2^ (≥27.5 kg/m^2^ if API).^24^ Coexisting chronic liver disease in addition to FLD was included. This was based on documentation of any other liver disease in the patient’s problem list, clinical notes, or laboratory evidence.

### Statistical Analysis

**Figure 1:**
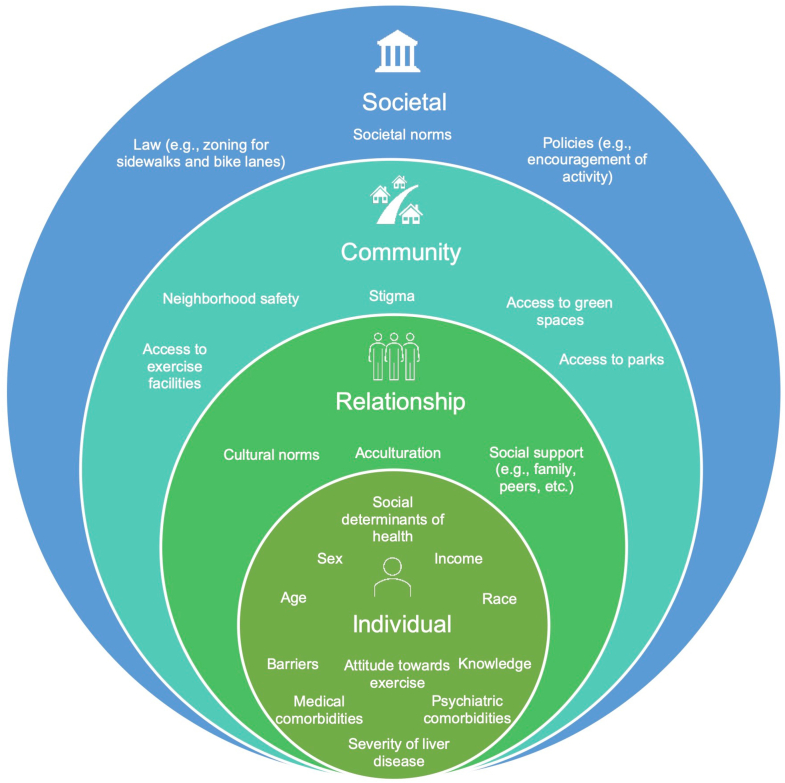
Socio-ecological model for uptake of physical activity in vulnerable population. This figure highlights the socio-ecological approach to understanding the inter-relationship between multiple factors associated with the uptake of physical activity. Individual, interpersonal, community, and societal factors play a role in behavioral changes.

After conducting an extensive literature review, a conceptual framework (Figure 1) was developed to describe individual, interpersonal, and society-level factors associated with the uptake of physical activity. This model is based on the National Institute on Minority Health and Health Disparities research framework, which highlights the importance of evaluating health outcomes from the lens of the socio-ecological model.^25^ Based on this framework, predictors of interest pertaining to the uptake of physical activity were set *a priori*. The primary outcomes of this study were physical activity both at baseline and change in physical activity after participation in an FLD education program.

Composite scores for domains and subdomains regarding patient knowledge, beliefs, and barriers were calculated from responses to questions designed to assess these factors as follows: (1) “FLD knowledge” score was computed as the number of correct responses to 10 questions (1 for correct, 0 for incorrect or do not know; max score 10); (2) scores for each “Beliefs about FLD” subdomain were determined by summing the numerical codes (1 or 0) assigned to the responses for corresponding questions, and coded as 1 for agree or positive response and 0 for disagree or a negative response [perceived severity of disease (max. score 4), treatment efficacy (max. score 2), self-efficacy to discuss FLD (max. score 1), perceived susceptibility to disease risk (max. score 2), and stigma (max. score 8)]; (3) “Barriers to lifestyle modification” score was determined by summing the numbers of barriers checked on a list of 22 choices (max. score 22); and (4) “Motivation to adhere to lifestyle modifications” was calculated by a 4-item score with 4 options included on a Likert scale response from “Not motivated at all” to “Extremely motivated” (max. score 16).

Descriptive analyses on study participant characteristics were performed to calculate frequency (percentage), mean (standard deviation), and median (interquartile range). Patient characteristics were compared by any physical activity (vs none) at baseline. Univariable and multivariable analyses were performed to assess the relationship between each predictor and physical activity level at baseline and at 6 months following education, respectively.

Based on the recent clinical guidelines, duration of physical activity was categorized into 3 groups: none (0 minutes/wk), insufficient (0–150 minutes/wk), or recommended (at least 150 minutes/wk).^17^ Intensity of physical activity was sorted into 3 categories: light (<3 metabolic equivalent of tasks [METs]), moderate (3–6 METs), and vigorous (>6 METs).^26^ The physical activity level (combined duration and intensity) was then categorized as optimal (moderate or vigorous activity of any duration), suboptimal (light activity of any duration), and none (no physical activity). Factors potentially associated with physical activity at baseline (none, suboptimal, or optimal) were assessed using multinomial logistic regression. A multivariable model was developed based on the results from the univariate models, along with clinical judgment and consideration for multicollinearity issues (|correlation coefficient ≥0.6 or variance inflation factor ≥4). Age, sex, race/ethnicity, and any variables with associations significant at the *P* = .1 level from the unadjusted models were included in the final model. To prevent overfitting, the number of covariate levels allowed in the multivariable model were restricted such that there were at least 5 events of the smallest outcome category per covariate level.^27^

Logistic regression analysis was used to assess factors associated with improvement (vs no change) in physical activity from baseline to 6-month follow-up. In addition, a subgroup analysis was performed to evaluate improvement in physical activity at follow-up among participants who reported “suboptimal” or “none” physical activity at baseline. Due to the small number of outcome events in this subgroup, logistic regression was used on the collapsed outcome categories “optimal” vs “suboptimal/none.”

Relative risk ratios (RRRs), odds ratios (ORs), and 95% confidence intervals (CIs) were reported from all models. Hypothesis tests were 2-sided, and the significance threshold was set to 0.05. Statistical analysis was performed using Stata (Version 16, StataCorp LLC, College Station, TX).

## Results

### Cohort Characteristics

Of the 314 patients enrolled, 262 participants underwent education and completed pre-education and posteducation surveys. Table 1 summarizes patient characteristics by self-reported physical activity. Compared to participants who reported any amount of physical activity by self-report, those who did not endorse physical activity were younger (median age 49 vs 55 years), more female (64% vs 56%), more likely to be Hispanic (66% vs 53%), and less likely to be Asian (18% vs 31%). In addition, a higher proportion was married or living with a partner (vs not married or living with a partner, 54% vs 47%), unemployed (57% vs 36%), had a larger household size (61% vs 54%), obese (75% vs 55%), reported a medical condition that limited their ability to exercise (20% vs 14%), and reported heavy and binge-drinking habits (30% vs 22%). At baseline, 74% of participants self-reported physical activity; 64% of participants exercised at light intensity (25% moderate and 12% vigorous) and 66% reached the recommended exercise duration of at least 150 minutes/wk.

**Table 1:** Cohort Characteristics

Characteristic	Patients who self-reported any physical activity (*N* = 231)^a^	Patients who self-reported no physical activity (*N* = 83)^a^
Age (median, IQR), y	55 (44–64)	49 (41–59)
Female sex [*N* (%)]	130 (56)	53 (64)
Race/ethnicity [*N* (%)]		
White	25 (11)	6 (7)
Asian/Pacific-Islander	71 (31)	15 (18)
Hispanic	122 (53)	55 (66)
Black	5 (2)	4 (5)
Other	8 (3)	3 (4)
Marital status [*N* (%)]	(N = 229)	–
Never married	57 (25)	17 (21)
Widowed/divorced/separated	64 (28)	21 (25)
Married/living with a partner	106 (47)	45 (54)
Birth country [*N* (%)]	(N = 229)	–
United States	41 (18)	14 (17)
Other	188 (82)	69 (83)
Primary language [*N* (%)]		
English	50 (22)	20 (24)
Spanish	111 (48)	49 (59)
Cantonese	40 (17)	7 (8)
Vietnamese	2 (1)	1 (1)
Other	28 (12)	6 (7)
Education level completed [*N* (%)]	(N = 228)	(N = 82)
High school education or less	143 (63)	51 (62)
More than high school	85 (37)	31 (38)
Annual income [*N* (%)]	(N = 225)	(N = 82)
<$10,000	57 (25)	18 (22)
$10,000–30,000	75 (33)	21 (26)
$30,000–50,000	24 (11)	9 (11)
>$50,000	12 (5)	4 (5)
Unknown/Declined to answer	57 (25)	30 (37)
Employment in the last year [*N* (%)]	(N = 227)	(N = 82)
Employed	82 (64)	35 (43)
Unemployed	145 (36)	47 (57)
Housing [*N* (%)]		
Stable housing	215 (93)	79 (95)
Homeless	2 (1)	0 (0)
Other/temporary housing	2 (1)	1 (1)
Unknown/declined to answer	12 (5)	3 (4)
Household size [*N* (%)]	(N = 227)	(N = 82)
1–2 members	104 (46)	32 (39)
3 or more members	123 (54)	50 (61)
Alcohol use in prior year [*N* (%)]	(N = 225)	(N = 81)
None/minimal	145 (64)	48 (59)
Moderate	30 (13)	9 (11)
Heavy/binge drinking	50 (22)	24 (30)
ALT (median, IQR), units/L	(N = 228) 47 (33–73)	(N = 81) 47 (34–96)
AST (median, IQR), units/L	(N = 227) 36 (28–56)	(N = 81) 40 (28–73)
BMI (median, IQR)^b^	(N = 226)	–
Normal/underweight	23 (10)	6 (7)
Overweight	78 (35)	15 (18)
Obese	125 (55)	62 (75)
Diabetes [*N* (%)]	88 (38)	35 (42)
Hypertension [*N* (%)]	101 (44)	38 (46)
Hyperlipidemia [*N* (%)]	115 (50)	34 (41)
Coexisting liver disease [*N* (%)]	40 (17)	11 (13)
Chronic hepatitis B	32 (14)	9 (11)
Chronic hepatitis C	8 (3)	2 (2)
Advanced fibrosis [*N* (%)]	28 (34)	61 (27)
Anxiety [*N* (%)]	21 (9)	9 (11)
Depression [*N* (%)]	48 (21)	21 (25)
Medical conditions limiting ability to exercise, by self-report [*N* (%)]	32 (14)	17 (20)

Average duration of physical activity per week categories: none/insufficient (<150 minutes/wk) and recommended (>150 minutes/wk).

Intensity of physical activity categories: light (<3 METs), moderate (3–6 METs), and vigorous (>6 METs).

ALT, alanine aminotransferase; AST, aspartate aminotransferase; BMI, body mass index; IQR, interquartile range.

a Unless otherwise specified in the table.

b Race-based BMI categories: normal weight <25 kg/m^2^ (<23 kg/m^2^ for Asian), overweight 25–29 kg/m^2^ (23–27.4 kg/m^2^ for Asian), and obese >30 kg/m^2^ (≥27.5 kg/m^2^ for Asian).

### Factors Associated With Physical Activity at Baseline

On univariable analysis (Table 2), females (vs males, RRR 0.43, 95% CI 0.22–0.83, *P* = .01), Hispanic individuals (vs non-Hispanic, RRR 0.46, 95% CI 0.24–0.90, *P* = .02), and those with type 2 diabetes (vs no diabetes, RRR 0.49, 95% CI 0.24–0.98, *P* = .04) had a reduced relative risk of reporting optimal physical activity (vs none) at baseline. Older age was associated with a higher relative risk of suboptimal physical activity (vs none) at baseline (RRR 1.39 per decade increase in age, 95% Cl 1.12–1.73, *P* = .003). In addition, presence of hyperlipidemia was associated with a higher relative risk of suboptimal physical activity (vs none) and advanced fibrosis was associated with a lower relative risk of optimal physical activity (vs none), but these did not reach statistical significance. On multivariable analysis adjusted for age, sex, and race/ethnicity, no variable was significantly associated with optimal physical activity, but older age remained significantly associated with reporting suboptimal physical activity compared to no physical activity. For every decade increase in age, the relative risk of suboptimal physical activity (vs none) increased by 37% (RRR 1.37, 95% CI 1.07–1.75, *P* = .01).

**Table 2:** Univariable and Multivariable Multinomial Logistic Regression Models for Physical Activity at Baseline (*N* = 307)^a^

Characteristic	Univariate model (vs none)						Multivariable model (vs none)					
	Suboptimal			Optimal			Suboptimal			Optimal		
	RRR	95% Cl	*P* value	RRR	95% Cl	*P* value	RRR	95% Cl	*P* value	RRR	95% Cl	*P* value
Age, by decade	**1.39**	**1.12**–**1.73**	**.003**	0.98	0.77–1.29	.98	**1.37**	**1.07**–**1.75**	**.01**	1.05	0.78–1.42	.73
Sex, female	0.96	0.55–1.67	.88	**0.43**	**0.22**–**0.83**	**.01**	1.05	0.57–1.92	.88	0.55	0.27–1.13	.11
Hispanic vs (non-Hispanic)	0.58	0.34–1.02	.06	**0.46**	**0.24**–**0.90**	**.02**	0.76	0.41–1.41	.38	0.58	0.27–1.21	.15
Marital status, ref never married (*N* = 305)												
Widowed/divorced/separated	1.12	0.52–2.40	.78	0.47	0.18–1.25	.13	–	–	–	–	–	–
Married/living with a partner	0.70	0.35–1.40	.32	0.70	0.32–1.53	.37	–	–	–	–	–	–
Preferred language, non-English (*N* = 306)	1.13	0.60–2.14	.70	0.89	0.42–1.91	.77	–	–	–	–	–	–
Education, high school or less (*N* = 297)	1.04	0.59–1.83	.89	0.89	0.45–1.74	.73	–	–	–	–	–	–
Annual income, less than $30,000 (*N* = 216)	1.26	0.58–2.74	.56	1.05	0.43–2.55	.91	–	–	–	–	–	–
Unemployment (*N* = 302)	1.25	0.72–2.15	.43	1.46	0.74–2.86	.28	–	–	–	–	–	–
Homelessness, unknown, or other	1.61	0.50–5.16	.42	1.30	0.31–5.39	.72	–	–	–	–	–	–
3+ members in household (*N* = 302)	0.77	0.45–1.34	.36	0.75	0.39–1.44	.39	–	–	–	–	–	–
Alcohol use, ref none (*N* = 300)												
Minimal/moderate	1.10	0.47–2.58	.83	1.10	0.39–3.10	.87	–	–	–	–	–	–
Heavy	0.63	0.33–1.18	.15	0.87	0.41–1.85	.72	–	–	–	–	–	–
BMI, ref normal (*N* = 303)												
Overweight	1.15	0.38–3.47	.80	1.66	0.47–5.82	.43	–	–	–	–	–	–
Obese	0.56	0.21–1.52	.26	0.40	0.12–1.30	.13	–	–	–	–	–	–
log2ALT (*N* = 302)	0.85	0.64–1.13	.263	0.80	0.56–1.14	.21	–	–	–	–	–	–
log2AST (*N* = 301)	0.82	0.61–1.11	.20	0.78	0.54–1.13	.19	–	–	–	–	–	–
Diabetes (*N* = 305)	1.02	0.60–1.75	.93	**0.49**	**0.24**–**0.98**	**.04**	0.89	0.50–1.56	.68	0.54	0.26–1.12	.10
Hypertension (*N* = 305)	1.14	0.67–1.94	.63	0.65	0.33–1.26	.20	–	–	–	–	–	–
Hyperlipidemia (*N* = 305)	1.66	0.97–2.83	.07	1.16	0.60–2.24	.66	1.31	0.74–2.33	.36	0.99	0.49–2.04	.99
Cardiovascular disease (*N* = 305)	1.19	0.35–3.97	.78	0.96	0.21–4.42	.95	–	–	–	–	–	–
Advanced fibrosis	0.82	0.47–1.45	.51	0.49	0.23–1.05	.07						
Renal disease (*N* = 305)	1.05	0.25–4.30	.95	0.45	0.04–4.10	.42	–	–	–	–	–	–

“No exercise” group served as the reference in both univariate and multivariable models.

Bold indicates *P* < .05.

BMI, body mass index; RRR, relative risk ratio.

a Unless otherwise specified in the table.

A sensitivity analysis was also performed with a stricter definition of physical activity goals defined as optimal (moderate or vigorous activity for at least 150 minutes/wk) and suboptimal (moderate/vigorous activity for less than 150 minutes/wk or light activity of any duration). Once again, on adjusted multivariable analysis, age emerged as the only factor significantly associated with suboptimal activity (vs none) (RRR 1.28, 95% CI 1.02–1.62, *P* = .04).

### Change in Physical Activity and Factors Associated With Improvement in Physical Activity at 6-month Follow-up After Educational Intervention

Immediately following education, patient’s knowledge, beliefs, and barriers to lifestyle modification were assessed. Table 3 summarizes composite scores for domains and subdomains regarding patient knowledge, beliefs, and barriers posteducation. Physical activity levels were then evaluated at 6 months following education. A higher proportion of participants reported engaging in physical activity following education compared to baseline. Of the 224 participants who reached 6-month follow-up, 91% (vs 74% at baseline) reported engagement in physical activity at 6 months following education; 58% were exercising at light intensity (32% moderate and 10% vigorous) and 61% were reaching the recommended exercise duration.

**Table 3:** Knowledge, Beliefs, and Barriers Scores Following FLD Education

Characteristic	Score (*N* = 262)^a^
FLD knowledge (mean, SD)	7.5 (±2.2) [max. score 10]
Perceived severity of disease (mean, SD)	2.5 (±1.0) [max. score 4]
Treatment efficacy score of 1 or more [*N* (%)]	243 (93%) [max. score 2]
Reported self-efficacy to discuss FLD [*N* (%)]	180 (69%)
Perceived susceptibility to disease risk [*N* (%)]	248 (95%)
Stigma (mean, SD)	2.5 (±1.8) [max. score 8]
Barrier to lifestyle modification (mean, SD)	2.7 (±2.9) [max. score 22]
Motivation to adhere to lifestyle modification (mean, SD) (*N* = 221)	2.7 (±1.4) [max. score 16]

SD, standard deviation.

a Unless otherwise specified in the table.

On univariable logistic analysis (Table 4), older age was associated with lower odds of improvement in physical activity after educational intervention (OR 0.77 per decade increase, 95% CI 0.61–0.97, *P* = .02) and Hispanic (vs non-Hispanic) race/ethnicity was associated with improvement (vs no change) in physical activity (OR 2.64, 95% CI 1.45–4.83, *P* = .002). Moreover, treatment efficacy score and self-efficacy to discuss FLD score were associated with lower odds of improvement (vs no change) in physical activity but these did not reach statistical significance. Due to the limitation on the number of variables allowed for multivariable analysis, 2-predictor models were performed. When including age and race/ethnicity in the model, Hispanic participants had significantly higher odds of reporting improvement in physical activity (vs no change) compared to their non-Hispanic counterparts (OR 2.36, 95% CI 1.27–4.39, *P* = .01) at 6-month follow-up. Age was no longer significantly associated with improvement (vs no change) in physical activity when controlling for race/ethnicity (OR 0.83, 95% CI 0.66–1.06, *P* = .1).

**Table 4:** Univariable Logistic Regression Models for Improvement in Physical Activity at 6-Month Follow-Up Post FLD-Education (*N* = 224)^a^

Characteristic	Univariate model (improvement vs no change)		
	OR	95% Cl	*P* value
Age, by decade	**0.77**	**0.61**–**0.97**	**.02**
Sex, female	1.04	0.58–1.86	.90
Hispanic, ref non-Hispanic	**2.64**	**1.45**–**4.83**	**.002**
Marital status, ref never married (*N* = 222)			
Widowed/divorced/separated	0.67	0.32–1.39	.28
Married/living with a partner	0.89	0.47–1.71	.73
Primary language, non-English (*N* = 223)	1.10	0.54–2.17	.83
Education, high school or less (*N* = 216)	0.87	0.48–1.58	.65
Annual income, less than $30,000 (*N* = 159)	0.68	0.29–1.60	.38
Unemployment (*N* = 219)	1.27	0.70–2.30	.43
Homelessness, unknown, or other	0.64	0.16–2.56	.53
3+ members in household (*N* = 220)	0.78	0.44–1.40	.41
Alcohol consumption, ref none (*N* = 219)			
Minimal/moderate	1.41	0.57–3.45	.46
Heavy	1.66	0.81–3.38	.17
BMI, ref normal (*N* = 221)			
Overweight	1.26	0.42–3.82	.68
Obese	1.61	0.57–4.52	.37
log2ALT (*N* = 221)	1.12	0.84–1.52	.42
log2AST (*N* = 220)	1.20	0.88–1.66	.25
Diabetes	1.07	0.60–1.90	.81
Hypertension	0.79	0.45–1.40	.43
Hyperlipidemia	0.70	0.39–1.23	.22
Cardiovascular disease	0.75	0.22–2.59	.65
Advanced fibrosis	0.900	0.48–1.67	.73
Renal disease	0.42	0.09–2.09	.29
FLD knowledge score (*N* = 212)	0.97	0.85–1.11	.67
Perceived severity of disease score (*N* = 212)	1.10	0.82–1.43	.56
Treatment efficacy score (*N* = 212)	0.53	0.27–1.04	.07
Self-efficacy to discuss FLD score (*N* = 212)	0.55	0.30–1.03	.06
Stigma score (*N* = 212)	0.93	0.79–1.09	.35
Barrier to lifestyle modification score (*N* = 212)	1.04	0.96–1.15	.32
Motivation to adhere to lifestyle modification score (*N* = 212)	0.86	0.70–1.10	.18

“No change” group served as the reference in both univariate and multivariable models.

Bold indicates *P* < .05.

BMI, body mass index; OR, odds ratio.

a Unless otherwise specified in the table.

A subgroup analysis was performed on the 170 participants who initially reported “suboptimal” or “none” physical activity at baseline. Alcohol use was the only significant factor on univariable or multivariable logistic regressions. On univariable analysis, compared to participants who did not drink alcohol, participants in the heavy/binge drinking category had a significantly higher odds of optimal activity level at 6-month follow-up (OR 2.04, 95% CI 1.08–3.84, *P* = .03). On multivariable analysis, when adjusting for age, the findings remained consistent (OR 1.98, 95% Cl 1.05–3.74, *P* = .04). Further exploration of this trend showed that 41% of participants in the heavy/binge drinking category (vs 28% of participants reporting no drinking) were exercising at an optimal level at 6-month follow-up after FLD education.

## Discussion

In this study, we showed that a formal FLD education program, in the liver specialty care setting, can result in an increased adoption of physical activity among a safety-net population. Additionally, certain groups, namely those with a history of heavy alcohol use and Hispanic individuals, were particularly receptive to this intervention in improving levels of their physical activity.

Previously documented correlates of physical inactivity include older age,^28^ female sex,^29^ non-White race,^30^ low socioeconomic status,^31^ and lower educational attainment.^32^ Our baseline assessment did not find any significant associations with socioeconomic status or education level; however, we noted a positive relationship between age and any physical activity, even if suboptimal. This may be related to older participants having more leisure time for physical activity in comparison to their younger counterparts, especially among socio-economically disadvantaged populations who are reliant on income and job attendance.^33^ There may, however, be a component of mobility limitation in the older population that explains the predilection for light activity of any duration (defined in our study as “suboptimal”) rather than moderate or vigorous activity observed in our study.

In terms of sex and race/ethnicity, females (vs males) had 57%, and Hispanic individuals (vs non-Hispanics) had 54%, lower relative risk of optimal physical activity (vs none) at baseline on univariable analysis. In addition, close to 32% of Hispanic participants in our study reported physical inactivity at baseline, mirroring the national statistic among this population.^34^ Prior studies have shown that barriers to activity in the Hispanic population may be related to immigration enforcement,^35^ concern with neighborhood safety,^35^ access to parks/gyms/green spaces,^36^ lack of social support,^37^ time limitations,^38^ and lack of motivation.^38^ Many of these barriers are structural in nature and reflect the disparities in healthcare access among this at-risk population. Nevertheless, Hispanic participants had a higher odds of making significant improvements in their physical activity habits, independent of age, after engagement in the FLD education program in comparison to their non-Hispanic counterparts. This is consistent with prior literature which has shown the efficacy of health promotion programs in facilitating the adoption of physical activity among this vulnerable population.^39^^,^^40^ Indeed, health education and empowerment programs have been shown to improve knowledge of disease state,^41^ adoption of other healthy lifestyle changes,^41^ and even clinical outcomes for racially diverse patients with chronic conditions like diabetes.^14^ Further research to assess the impact of an FLD health education program on clinical end points (eg, anthropometric measurements, liver function tests, and liver steatosis/fibrosis grade) are currently under investigation in our study population.

A novel finding of our study was that participants who reported heavy/binge drinking habits had an increased odds of “optimal” levels (vs “suboptimal/none”) of physical activity following education. Adoption of lifestyle modification suggests that simple educational interventions are effective in improving physical activity levels and in turn, enhancing cardio-metabolic health in this patient population, which may have a positive impact on their liver disease progression.^5^^,^^6^ In fact, exercise has been shown to improve body composition and reduce hepatocyte apoptosis in overweight patients who consume alcohol.^42^ The treatment for ALD is alcohol cessation/reduction; however, only an estimated 7% of adults with alcohol use disorder engage in treatment annually.^43^ While significant effort should be directed toward the treatment of alcohol use to achieve abstinence, optimizing metabolic health through lifestyle modification could provide these patients with measures to mitigate further liver damage.

Our study had several limitations. Although the stratification of physical activity into intensity and duration was representative of recent society-based guidelines, we were not able to ascertain whether participants were exercising at their self-reported intensity throughout the entire duration. This study also took place at a single center, limiting the generalizability across other healthcare settings. Self-report, recall bias, and response bias were other limitations noted in the context of any survey-based study. However, a major strength of this study was the survey of a diverse vulnerable patient population at increased risk for FLD burden and the participation of patients with both NAFLD and ALD. Our study population is typically under-represented in medical research and further enriches the field of knowledge in minority health and healthcare disparity. Furthermore, the surveys implemented in this study covered a comprehensive assessment for social determinants of health.

In summary, we found that formal FLD education led to increased uptake of physical activity within a vulnerable population. Marginalized populations often encounter obstacles to equitable care because of intersecting individual-level, community-level, and society-level factors. Our study demonstrates the positive impact of patient-centered approaches in promoting uptake of physical activity, importantly among Hispanic individuals and those reporting heavy alcohol use, populations historically at risk for experiencing health inequities.
